# Cannabinoid and Serotonergic Systems: Unraveling the Pathogenetic Mechanisms of Stress-Induced Analgesia

**DOI:** 10.3390/biomedicines12010235

**Published:** 2024-01-19

**Authors:** Hristina Nocheva, Nikolay Stoynev, Vlayko Vodenicharov, Dimo Krastev, Nikolay Krastev, Milka Mileva

**Affiliations:** 1 Department of Physiology and Pathophysiology, Medical Faculty, Medical University, 2 Zdrave Str., 1431 Sofia, Bulgaria; hndimitrova@medfac.mu-sofia.bg (H.N.); nstoynev@medfac.mu-sofia.bg (N.S.); 2 Department of Epidemiology and Hygiene, Medical Faculty, Medical University, 2 Zdrave Str., 1431 Sofia, Bulgaria; v.vodenicharov@medfac.mu-sofia.bg; 3 Department of Anatomy and Physiology, South-West University “Neofit Rilski”, Blagoevgrad, 66, Ivan Mihaylov Str., 2700 Blagoevgrad, Bulgaria; dimo_krustev@mail.bg; 4 Department of Anatomy, Faculty of Medicine, Medical University, 2, Zdrave Str., 1431 Sofia, Bulgaria; dr.krustev.dm@gmail.com; 5 Institute of Microbiology “Stephan Angeloff”, Bulgarian Academy of Sciences, 26, Acad. Georgi Bonchev Str., 1113 Sofia, Bulgaria

**Keywords:** pain perception, cannabinoid receptor CB1, 5-HT receptor 1A, cold stress-induced analgesia, stress-response

## Abstract

The perception of „stress” triggers many physiological and behavioral responses, collectively called the stress response. Such a complex process allows for coping with stress and also triggers severe pathology. Because of the multidirectional effect of stress on the body, multiple systems participate in its pathogenesis, with the endogenous cannabinoid and the serotoninergic ones among them. These two systems also take part in the pain perception decrease, known as stress-induced analgesia (SIA), which can then be taken as an indirect indicator of the stress response. The aim of our study was to study the changes in cold SIA (c-SIA) resulting from the exogenous activation of cannabinoid receptor type 1 (CB1) and 5-hydroxytryptamine (5-HT, serotonin) receptor type 1_A_ (5-HT1A). Various combinations of agonists and/or antagonists of CB1 and 5-HT1A, before or after 1 h of cold exposure, were applied, since we presumed that the exogenous activation of the receptors before the cold exposure would influence the pathogenesis of the stress response, while their activation after the stressful trigger would influence the later development. Our results show that the serotonergic system “maintained” c-SIA in the pre-stress treatment, while the cannabinoids’ modulative effect was more prominent in the post-stress treatment. Here, we show the interactions of the two systems in the stress response. The interpretation and understanding of the mechanisms of interaction between CB1 and 5-HT1A may provide information for the prevention and control of adverse stress effects, as well as suggest interesting directions for the development of targeted interventions for the control of specific body responses.

## 1. Introduction

As things have become increasingly complex and hectic, stress seems to be a ubiquitous aspect of life. Confrontation with adverse circumstances, perceived as „stress”, triggers in both humans and animals a cascade of intricate physiological and behavioral responses, collectively referred to as the stress response. At the heart of such a response, a range of coordinated events orchestrates a network of afferent and efferent projections, starting with the activation of the autonomic nervous system (ANS) and the hypothalamic-pituitary–adrenal (HPA) axis, and culminating in the release of glucocorticoids from the adrenal cortex. The ANS is responsible for the immediate reactions to the stressor—the activation of the sympathetic nervous system underlines the “fight or flight” response, enabling the body to defend itself, while the parasympathetic nervous system aims at restoring the balance when the stressor has been answered. The hypothalamus is a crucial player, acting as a central coordinator of the stress response. The activation of the HPA finally leads to cortisol release, which exerts widespread effects on metabolism, immune function, and inflammation. Stress changes the biochemistry of the brain, involving other specific areas, such as the limbic system and brainstem nuclei. Glucocorticoid feedback along the HPA axis is regulated at the level of the hypothalamus by a diverse group of afferent and efferent projections to the limbic lobe of the brain, brainstem nuclei, and projections along the spinal cord [1].

The main evolutionary purpose of the stress response is to provide an opportunity for the organism to optimally cope with a specific adverse situation, increasing its adaptation and the chance to survive. But this complex process, driving the homeostasis to a thoroughly different level, can also trigger specific (stress-)induced pathology [2,3]. A complex series of biochemical reactions disrupt the body’s homeostasis leading to changes in behavioral responses. A growing number of studies in this direction indicate that a stressful lifestyle (acute or chronic exposure to stress) is associated with increased arterial pressure, endothelial dysfunction, disturbances in the lipid profile, and metabolic deviations, which in turn are the basis of significant social pathology: leading causes of mortality (such as cardiovascular disease, diabetes, cancer), decreasing quality of life (obesity), or other unfavorable consequences (reproductive problems).

Given the negative consequences of stress, many studies have tried to reveal its underlying pathogenetic mechanisms. Understanding the molecular mechanisms of the stress response is crucial since that would make it possible to determine specific practical approaches and strategies to limit or at least mitigate the pathological effects. The difficulty in studying stress is largely related to the subjective nature of the experience. The perception of a specific situation such as „stress“ by the specific individual depends on factors such as attitude, value system, motivation, and others, which can hardly be objective; the individual perception of the predictability and controllability of the stressful situation also plays a role [4]. In order to track the influence of specific impacts on the stress-reaction, the use of an objective indicator is required that can be relatively easily measured and serve to objectify changes over time. During the stress response, many physiological parameters of the organism change with the aim of optimal adaptation to the specific situation, adequate response, and the possibility of survival. In this context, the perception of pain, which is basically defined as protective (since it includes reflexes aimed at preserving the health and life of the individual), in the specifics of the stress response, appears unfavorable. A kind of paradox arises—pain perception would limit the organism’s ability to overcome stress. Therefore, it seems logical that pain perception decreases during the stressful situation, thus eliminating its paralyzing effect on the body.

The first information about the decrease in pain perception in stressful situations was provided by Beecher, who observed wounded soldiers during the World War II. He noted that wounds, which under other circumstances were felt as very painful, caused a weak sensation of pain [5,6,7]. In fact, this built-in mammalian pain-suppressing response has a defensive purpose—making it possible to focus more effectively on the stressful (fearful) stimulus, thus better coping with the stress [8]. SIA is a complex process, although endogenous opioids play a key role in mediating endogenous analgesia [9], several mediating systems have also been proved to be involved [10]. As for the anatomical substrate of SIA, some subcortical areas, such as the periaqueductal gray, the amygdala, and the rostral ventromedial medulla, seem critical for the descending inhibitory pain pathways [10,11,12]. The dependence of SIA on stress itself allows an increase in the pain threshold during the stress-dominated period to be taken as a relatively objective indicator of the body’s stress reaction.

Given the multidirectional effect of stress on the body, multiple systems are implicated in its pathogenesis, and one in particular has been demonstrated to participate in the mechanism of SIA [13]. In recent decades, the endocannabinoid system (ECS) has been the focus of many studies due to its participation in both physiological and pathophysiological reactions [14,15]. The ECS is a neuromodulatory system consisting of (i) a complex network of G-protein-coupled cannabinoid receptors type 1 (CB1) and 2 (CB2) [15], widely distributed in the central and peripheral nervous systems [16]; (ii) their endogenous ligands—endocannabinoids: anandamide (N-arachidonoyl ethanolamine, AEA) and 2-arachidonylglycerol (2-AG); and (iii) an enzyme system engaged in their biosynthesis and subsequent metabolisms [15]. However, there may also be additional “players” such as the transient receptor potential vanilloid 1 (TRPV1) [17] and several putative CB1 receptor antagonist peptides [15]. The cannabinoid CB1 receptors are highly expressed in several limbic brain regions (i.e., hippocampus, amygdala, prefrontal cortex), and involved in the HPA axis [18,19] and adrenal gland regulation [20]. The CB2 receptors have been detected in glial cells, and, to a much lesser extent, in neurons of several brain regions such as the amygdala, hippocampus, cerebral cortex, hypothalamus, and cerebellum [21,22]. For the moment, the overall evidence indicates the pivotal role of CB1, and not CB2, in HPA axis regulation following stress exposure [23,24].

As a lipid signaling system whose components are expressed widely across the body, the ECS plays a key role in the regulation of a wide array of physiological processes including metabolism, mood, motor function, appetite, cardiovascular control, gastrointestinal tract function, developmental biology, cell fate, immune and inflammatory response, endocrine function, neurotransmission, and pain [25]. It appears that the ECS plays an important role in the regulation of stress-related behavior [26], with its role appearing to be aimed at modulating the stress-response in order to “spare” the organism. The system continues to be the focus of many studies in the attempt to “rehabilitate” exogenous cannabinoids and enable their wider use given their many positive effects on the body. Research mostly focuses on several directions: (a) evaluating the pharmacology of cannabinoids and endocannabinoid system modulators; (b) evaluating cannabinoids’ effects in different animal models of pathological or injury-related persistent pain; (c) describing the pharmacokinetics of cannabinoids in humans. Some cannabis-based medicines (CBMs) have proven to be efficient in reducing chronic pain [27,28]. In addition to pain, the therapeutic use of cannabis reduces stress, distress, and anxiety in both experimental animals and humans [29]. The results from animal studies have shown that the pharmacological blockade of CB1 receptors alters stress-induced behavior [30,31] and models conditioned fear responses [32]. The pharmacological enhancement of ECS signaling, by the blockade of endocannabinoids’ metabolism and/or uptake, reduces stress-related behavior and facilitates the extinction of stress-conditioned responses [33]. At the same time, proof exists that the chronic use of cannabis has the opposite effect, leading to an increase in mental and somatic symptoms, including anxiety and panic attacks [29,34]. It is the observed adverse side effects that fuel the reserves of the ECS’s opponents.

In the last decade, our team has also focused on the ECS during stress. It is not the only system involved in the stress response: behavioral responses to stress are similar in humans and animals, and this complex response includes different neurotransmitters—catecholamines, serotonin, dopamine, dynorphin, 5-HT, acetylcholine, nitric oxide, and, of course, endocannabinoids [2]. This encouraged us to evaluate the interactions of ECS with other mediator systems—adrenergic [35] and nitric oxide [36], as well as the joint effects of cannabinoids with the Tyr-MIF-1 family of peptides [37]. Our observations substantiated the need for further investigation into ECS signaling under various stressogens, and ECS’s interrelation with the serotonergic system seemed to be a promising candidate [38,39].

Serotonin (5-hydroxytryptamine, 5-HT) is one of the key neurotransmitters involved in a wide variety of behavioral and cognitive responses. 5-HT-releasing neurons are vastly distributed in the central nervous system, which is the primary target of nociceptive information. Such neurons can regenerate and their activation under various stressful conditions is associated with depression, anxiety, and cognitive impairment. Serotonin is released in association with pain-related behaviors, manifesting both pro- and antinociceptive effects [40,41,42].

The investigations of Marks et coauthors (2009), as well as Chae et al. (2020), also support the theory of the interaction between cannabinoid and serotonergic systems in the brain [43,44,45]. Additionally, there are shreds of evidence indicating that interplays between the two systems are also involved in the stress-response development [46]. SIA is a relatively easy indicator to be determined. On the other hand, it could be used to objectify the stress response, as numerous studies show the relationship between the two of them. In this regard, there is evidence that certain parameters’ changes during stress invariably and specifically affect pain sensitivity and, accordingly, cause SIA—e.g., the increase in endogenous opioid levels [47], the activation of the sympathetic nervous system [48], or the potentiation of the descending control of spinal nociception [49]. Taking the level of SIA as an indirect indicator of the degree of the stress response, we decided to evaluate the changes in the pain thresholds of rats exposed to one hour of cold environment.

The healing effects of cold on the body were already known to the ancient Egyptians and Greeks. They used cold water immersion to treat various ailments and pain symptoms [50].

In modern clinical practice for pain reduction, cryotherapy is a widely used modality for pain relief, which is practiced in a wide range of medical fields and produces analgesic effects. Specialist clinicians classify it as the so-called non-pharmacological approaches to achieve pain control and an analgesic effect in which the threshold of pain sensitivity is increased [51,52].

In a previous study of our team, we evaluated the effects of cannabinoids and the nitric oxide-ergic system on the modulation of stress response before and after restraint stress—the results showed interesting differences in the effect of cannabinoid-nitric oxide interaction on restraint-SIA before and after stress [53]. Such findings encouraged us to hypothesize that exogenous factors would have different effects if administered before or after the stressful impact: in the first case they would be involved in the pathogenesis of the stress reaction, while in the second in its modulation. On the other hand, both the endogenous cannabinoid and the serotonergic systems are known to be involved in the body’s stress-response but also take part in SIA. Acute stress has been proved to exert an analgesic effect by activating the serotonergic system [54]; 2-arachidonoylglycerol and anandamide increase in the midbrain after acute stress has been demonstrated, pointing at an endocannabinoid mechanism involved in stress-induced analgesia [13]. Considering the evidence for the involvement of the two systems in the stress response and the development of cold SIA, in the present study, we aimed to investigate the interaction between cannabinoids and the serotoninergic system through the exogenous activation of cannabinoid receptor type 1 (CB1) and 5-hydroxytryptamine (5-HT, serotonin) receptor type 1A (5-HT1A). To further refine the involvement of each of the systems in the reported effects, we provided additional treatments with the appropriate antagonists of both receptor types. The analysis of the combined results of the agonists’ effect, on the one hand, compared with the results obtained after antagonizing one receptor type with the simultaneous activation of the other, on the other hand, would allow us to better specify the importance of each of the systems for their joint effect.

The benefit of research on the mechanisms of stress can be seen in several directions. To begin with the medical and psychological influences of post-traumatic disorders, depressive, anxiety disorders, etc., preventive strategies could also be developed based on interventions to mitigate stress impact. Specifically, regarding the ECS, investigating its involvement in the stress response and the potential benefits of its activation or suppression may provide interesting directions for the development of targeted interventions and medications that modulate specific body responses.

## 2. Materials and Methods

### 2.1. General Study Design

The aim of this study was to determine the joint effect of the cannabinoid and the serotonergic systems on cold SIA. The effect of both systems was followed by administration of cannabinoid (CB1) and serotonin (5-HT1A) receptor agonists before and after one hour of cold stress.

Further clarification of the degree of involvement of each of the systems in the reported effect was achieved by injecting the animals with combinations of an agonist of one receptor and an antagonist of the other, again before and after the stressogenic impact.

Cold stress method has been described long ago, and among the first to use it were E. Zeisberger [55] and Z. Wiesenfeld and R. G. Hallin in 1981 [56]. The method has evolved into cold water immersion, cold water swim, repetitive cold stress, chronic cold stress. In our experiments, we aimed to induce not cold stress itself but cold stress-induced analgesia, and for such purposes, we needed an acute stress method. It should also be a stress method that is easy to induce and is effective at the same time to activate the HPA axis without causing permanent physical or psychological disorders in the experimental animals. Our previous experiments [36,57] showed that one hour of cold environment (4 °C) exposure provoked stress analgesia—experimental animals’ paw pressure thresholds were statistically higher than control animals’ ones. In addition, substantial studies have confirmed the effects of cold stress on memory and behavior, as well as its implication in some cognitive changes and anxiety disorders [58].

To determine analgesia, we chose the Randal Selitto Paw Pressure test method—it allows repeated determination of the pain threshold without negative consequences for the experimental animals, as well as without causing significant discomfort, which makes it suitable according to the ethical criteria for working with laboratory animals [59].

### 2.2. Animals

Adult male Wistar rats, 250–300 g body weight (BW), were kept in plastic cages under a 12 h light:12 h darkness cycle (light onset at 08.00 h), at 24 ± 1 °C; a standard diet and tap water were available ad libitum [60]. All experimental protocols (regarding the number of animals in the experimental groups and the respective treatments) were approved by our institutional animal care committee—the Bulgarian Food Safety Association (BFSA)—Permission Protocol № 314/06.10.2021.

### 2.3. Methodology

Since we hypothesized that the interaction between CB1- and 5HTA1-agonists could have a different outcome if the receptors were activated before or after the stressful impact, the animals were treated with a combination of CB1- and 5-HT1A-agonists before or after cold environment exposure. An eventual decrease in pain thresholds would point to an anti-stress effect, while the increase in the pain thresholds should be regarded as an indicator of increased activity in the body’s stress systems.

In the pre-stress experimental set-up, the measurement of pain thresholds began 10 min after the end of the cold exposure.

In the post-stress experimental set-up, the measurement of pain thresholds started 10 min after the injection of the substances.

### 2.4. Experimental Groups 

Group 1 (Controls)—the animals (*n* = 8) in this group were injected with 1 mL of saline;

Group 2 (AEA+DPAT+1 h CS)—the animals (*n* = 8) in this group were injected with CB1-agonist (anandamide, AEA) and 5-HT1A- receptors’ agonist (8-Hydroxy-DPAT hydrobromide, DPAT) BEFORE being subjected to 1 h of cold stress;

Group 3 (1 h CS+AEA+DPAT)—the animals (*n* = 8) in this group were injected with agonists of both receptors (AEA and DPAT) AFTER being subjected to 1 h of cold stress;

Group 4 (AEA+NAN+1 h CS)—the animals (*n* = 8) in this group were injected with CB1 receptors’ agonist AEA and 5-HT1A receptors’ antagonist (NAN-190 hydrobromide, NAN) BEFORE being subjected to 1 h of cold stress;

Group 5 (1 h CS+AEA+NAN)—the animals (*n* = 8) in this group were injected with CB1 receptors’ agonist AEA and 5-HT1A receptors’ antagonist NAN AFTER being subjected to 1-h of cold stress;

Group 6 (DPAT+AM+1 h CS)—the animals (*n* = 8) in this group were injected with the 5-HT1A receptors’ agonist DPAT and CB1 receptor’s antagonist AM251 BEFORE being subjected to 1 h of cold stress;

Group 7 (1 h CS+DPAT+AM)—the animals (*n* = 8) in this group were injected with of the 5-HT1A receptors’ agonist DPAT and CB1 receptors’ antagonist AM251 AFTER being subjected to 1 h of cold stress.

### 2.5. Acute Model of Cold Stress

Acute cold stress was induced by placing the animals at a low environmental temperature (4 °C) for 1 h. During the time of cold exposure, no food and water were allowed; the rats could move freely, allocated in individual cages without sawdust.

### 2.6. Drugs 

All the drugs were purchased from Sigma (Sigma Chem. Co., St. Louis, MO, USA). The CB1-agonist N-arachidonoyl-ethanolamine (AEA, 1 mg/kg BW); the CB1-antagonist N-(Piperidin-1-yl)-5-(4-iodophenyl)-1-(2,4-dichlorophenyl)-4-methyl-1H-pyrazole-3-carboxamide (AM251, 1.25 mg/kg BW); the 5HT1A-agonist (R)-(+)-8-Hydroxy-DPAT hydrobromide (DPAT, 1 mg/kg BW); and the 5HT1A-antagonist NAN-190 hydrobromide (NAN, 1 mg/kg BW), dissolved in vehicle [61,62] were intraperitoneally administered in different combinations before or after stress exposure.

### 2.7. Nociceptive Test

Paw-pressure test (PP; Randall–Selitto test): The changes in the mechanical nociceptive thresholds of the rats were measured using an analgesimeter (Ugo Basile, Gemonio, Italy). The pressure was applied to the rat hind-paw and the pressure required for eliciting a nociceptive response, such as a squeak or struggle, was taken as the mechanical nociceptive threshold (paw-pressure thresholds, PPT—represented in arbitrary units, AU, according to the scale of the analgesimeter). A cut-off value of 500 g was observed to prevent damage in the paw [63].

### 2.8. Statistical Analysis

Results were statistically assessed using a General Linear Model for repeated measures (mixed model ANOVA), and one-way analysis of variance (ANOVA at each time point followed by Newman–Keuls post hoc comparison test. Values were presented as mean ± S.E.M and *p* < 0.05 was considered to indicate statistical significance.

## 3. Results

In the present study, we investigated the interaction between exogenously administered cannabinoid (AEA) and serotonin receptor (DPAT) agonists, and their joint effect on cold stress-induced analgesia, determined by measuring the paw pressure threshold (PPT).

The co-administration of the substances was before or after exposure of the experimental animals to one-hour of cold (4 °C).

### 3.1. Antinociceptive Effect of AEA and DPAT before and after 1 h of Cold Exposure

For our experiment, we chose the doses as follows: the CB1-agonist N-arachidonoylethanolamine (anandamide, AEA, 1 mg/kg BW); the CB1-antagonist N-(Piperidin-1-yl)-5-(4-iodophenyl)-1-(2,4-chlorophenyl)-4-methyl-1H-pyrazole-3-carboxamide (AM251, 1.25 mg/kg BW); the 5HT1A-agonist (R)-(+)-8-Hydroxy-DPAT hydrobromide (DPAT, 1 mg/kg BW); and the 5HT1A-antagonist NAN-190 hydrobromide (NAN, 1 mg/kg BW), dissolved in the vehicle.

**Figure 1:**
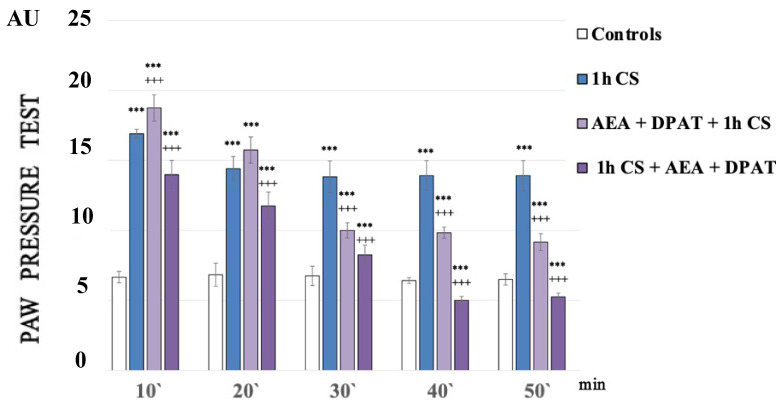
Effect on cold-SIA after administration of CB1 agonist (anandamide, AEA) and 5HT1A-agonist DPAT before or after stress exposure. Pain thresholds are presented as mean values ± S.E.M. in arbitrary units (AU). *** *p* < 0.001, vs. controls; +++ *p* < 0.001. AEA—exogenously administered anandamide; DPAT—5-HT1A-agonist; 1 h CS—1 h of cold stress.

Figure 1 shows the paw pressure thresholds (PPT) of the experimental animals after 1 h of exposure at 4 °C (1 h of cold stress, 1 h CS). In the 1h CS group, we observed an increase in PPT values compared with those of the control animals. One-way ANOVA showed a significant effect—*p*-values were <0.00001 (F = 2749.61972 on the 10th min; F = 1375.5814 on the 20th min; F = 1962.33333 on the 30th min; F = 2373.71795 on the 40th min) for the whole time estimated (Figure 1).

At the very beginning of the experiments, the control values of the pain thresholds were determined using the paw pressure method (our long-term practice shows that, if we work with properly handled animals, there is no statistically significant difference between the paw pressure thresholds, PPT, of animals injected with a physiological solution and intact animals).

One hour of cold exposure (1 h of cold stress, 1 h CS) produced a sustained and statistically significant increase in paw pressure thresholds (PPT) in the experimental group compared with the control one. The results of the experiments were analyzed using one-way ANOVA.

A statistically relevant potentiation of cold-SIA (c-SIA) was observed at the 10th min after AEA and DPAT pretreatment (*p* = 000021, F = 38.98676), while a decrease in PPT followed the administration of the same combination (AEA+DPAT) after stress exposure (Figure 1).

### 3.2. Effects of Agonist/Antagonist Co-Administration before and after Cold Exposure on Cold-SIA

To better elucidate the contribution of each of the two systems to the effects described, we chose an approach in which each one of the agonists was co-administered with the antagonist of the other receptor.

**Figure 2:**
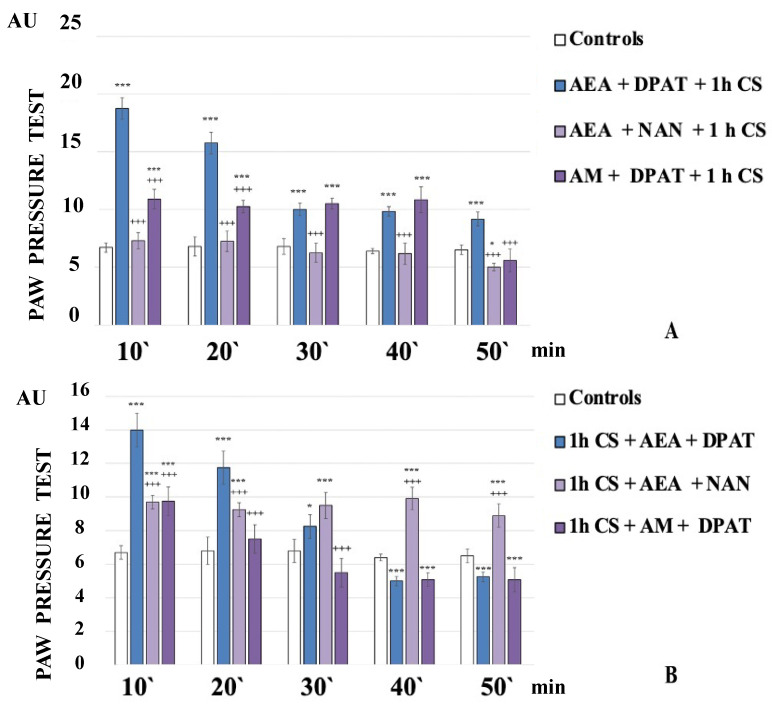
CB1- or 5HT1A-antagonization before (**A**) and after (**B**) stress exposure—effect on 1 h cold-SIA. Pain thresholds are presented as mean values ± S.E.M. in arbitrary units (AU). *** *p* < 0.001, * *p* < 0.05 vs. controls; +++ *p* < 0.001 vs. AEA+DPAT+1 h CS (**A**)/1 h CS+AEA+DPAT (**B**). AEA—exogenously administered anandamide; DPAT—5-HT1A-agonist; NAN—5-HT1A-antagonist; AM—CB1-antagonist; 1 h CS—1 h of cold stress.

The administration of the CB1 agonist AEA together with the 5HT1A-antagonist NAN before exposure to stress completely abolished the development of c-SIA. The obtained results showed that the PPT of the experimental animals were similar to the controls and even showed a tendency towards hyperalgesia at the 50th minute of the experiment (AEA+NAN+1 h CS, Figure 2A).

AEA+NAN-administration after 1 h CS led to a constant level of c-SIA. The PPT of 1 h CS+AEA+NAN-animals were lower than the 1 h CS+AEA+DPAT-animals’ ones at the 10th and 20th min but they exceeded them from the 30th min until the 50th min of the experiment (Figure 2B).

The administration of 5HT1A-agonist DPAT along with the CB1-antagonist AM251 before stress exposure led to a constant level of c-SIA for the first 40 min, followed by a brisk decrease at the 50th min of the experiment. The PPT of AM+DPAT+1 h CS-animals were lower than AEA+DPAT+1 h CS-animals’ ones at the 10th and 20th min, while at the 30th and 40th min, they were comparable to them (Figure 2A).

AM+DPAT-administration after 1 h CS decreased PPT at the 10th min compared with 1 h CS+AEA+DPAT-animals’ ones, with no c-SIA detected at the 20th min, and a tendency toward hyperalgesia from the 30th min to the end of the experiment (1 h CS+AM+DPAT, Figure 2B).

The analysis of the data obtained from the different experimental setups allowed us to confirm our hypothesis about the joint effect of the exogenous activation of the cannabinoid and serotonergic systems. The results obtained pointed that the two systems impacted on c-SIA, decreasing it, but they participated differently in the pathogenesis of the stress reaction and in the modulation of an already activated stress response of the body. In a more general context, the results should be considered in terms of the individual and joint importance of the systems in the body’s stress response, pain perception, and the possibility of including them in therapeutic schemes approfittating of their positive influence.

## 4. Discussion

The exogenous manipulation of cannabinoid and serotonin receptors by means of agonists and antagonists allowed us to draw different conclusions regarding the joint effect of the two systems on c-SIA.

In first place, cold exposure led to statistically higher PPT in experimental animals compared with control ones, allowing us to conclude that stress-analgesia was induced. The results were concordant with the literature data about cold stress as a factor inducing stress analgesia, including our previous findings [36,64]. 

**Figure 3:**
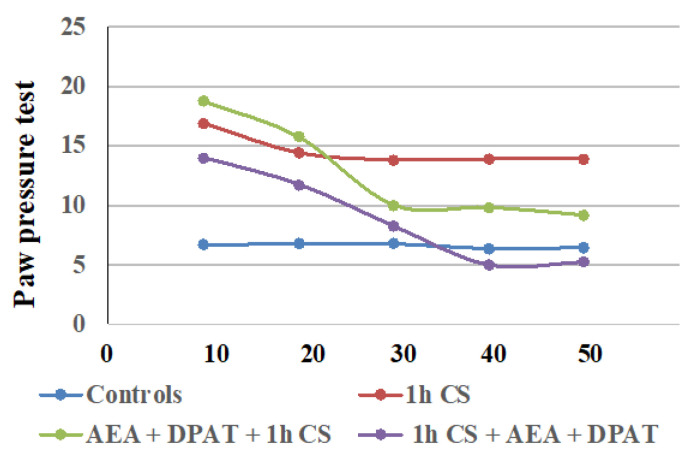
Effect on cold-SIA after administration of CB1 agonist (AEA) and 5HT1A-agonist DPAT before or after stress exposure—the results are presented as tendencies over time. AEA—exogenously administered cannabinoid; DPAT—5HT1A-agonist; AM—CB1-antagonist, CS—1 h of cold stress.

In second place, we found that the exogenous administration of CB1- and 5HT1A-agonists together, before or after stress, generally influenced c-SIA in rats, and the changes in PPTs differed before and after stress exposure. Our findings are summarized in Table 1, and additionally illustrated in Figure 3.

**Figure 4:**
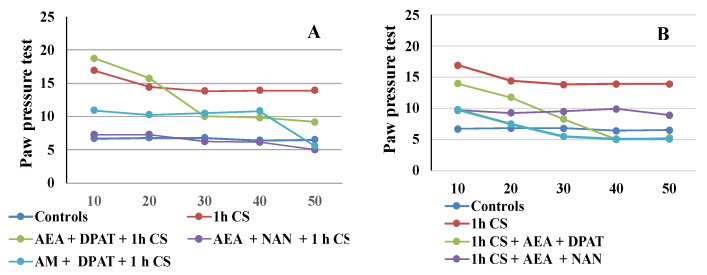
CB1- or 5HT1A-antagonization before (**A**) and after (**B**) cold stress exposure and effect on 1 h cold-SIA—the results are presented as tendencies over time. AEA—exogenously administered anandamide; DPA—5-HT1A-agonist; NAN—5-HT1A-antagonist; AM—CB1-antagonist, 1 h CS—1 h of cold stress.

The results are consistent with the literature data on the involvement of ECS in analgesia. At the supraspinal level, cannabinoids have been proved to exert analgesic action in the periaqueductal gray [65,66], the thalamus [67], the rostral ventromedial medulla [68,69], and the amygdala [32,70]. Cannabinoids suppress behavioral responses to noxious stimulation and decrease nociceptive processing through the activation of cannabinoid CB1 and CB2 receptor subtypes [71]. At the spinal level, an endocannabinoid modulative effect on nociception has been documented in behavioral [72,73], electrophysiological [74,75,76], and neurochemical [77,78] studies. The endocannabinoid analgesic effect has also been proved at the peripheral level in several animal models [79,80,81]. Such multi-level involvement of the ECS in the mechanisms of analgesia probably accounts for the observed maintenance of some level of analgesia until the end of the follow-up period in the pre-treatment trials. The effects from cannabinoids and serotonin agonists’ administration before cold exposure imply that the corresponding receptors participate in a specific way in the mechanisms of development (i.e., in the pathogenesis) of the body’s stress response. Since we assume that the changes in the PPTs of animals exposed to stress, compared with those of animals without stress (controls), are an indirect indicator of the level of the stress-reaction of the organism, we could conclude that the exogenous administration of both agonists modulates the stress-response with an initial activation followed by a moderate decrease in activity. The results also support the idea that the serotonergic system is relatively more important than the ECS in “maintaining” c-SIA in pre-treated animals, while in the case of post-treatment, analgesia depends, to a relatively higher degree, on the activity of the cannabinoid system. Our conclusions have been additionally illustrated in Figure 4A,B.

The statistically significant decrease in c-SIA level described in our study after the exogenous administration of both cannabinoid- and 5-HT1A-receptors’ agonists after the stressor, could be explained by the endocannabinoids’ modulation of the neuroendocrine function through the HPA axis [82]. The decrease in its activity is probably important for the better adaptation and survival of animals when exposed to stressful stimuli. In the conditions where the experimental animals have already been exposed to cold, and therefore c-SIA has already been induced, the subsequent introduction of the combination of cannabinoid and serotonin receptors’ agonists suggests that the interaction between both receptors modulates c-SIA—decreasing it, but not thoroughly abolishing it.

Our results indicate that the interaction between the two systems has opposing effects before and after the stressogenic impact. Moreover, the activation of serotonin receptors prior to stress appears to be a necessary condition for the onset of c- SIA as well as its duration, thus the interaction follows the “all or nothing” principle. Conversely, once c-SIA has already been induced, antagonizing the 5HTA1-receptors contributes to its duration. Our research shows that interactions between mediator systems differ in non-stress and post-stress conditions, and the outcome of these different interactions differentially affects the stress response itself. It is logical to expect that not only the systems we have investigated are subject to different relationships, but as are all other mediator systems. This suggests that the prophylactic and therapeutic protocols of influencing the stress-response, resp. stress-induced pathology, should take such differences into account. On the other hand, from the obtained results, and in particular from the overtime tendencies illustrated in Figure 3 and Figure 4, it can be seen that the effect of the interactions between the systems varies over time. This is logical, insofar as the timely and effective activation of stress-response mechanisms favors adaptation, but at the same time, their timely shutdown favors the restoration of balance and homeostasis. Such logic supports the multidirectional effect we have demonstrated of the interaction between endocannabinoids and the serotonin system.

A known limitation in the interpretation of the in vivo effects described above is the lack of indications about specific changes in receptors’ expression. In a previous study, we performed an in vivo determination of the analgesic activity of administered substances coupled with the in vitro immunohistochemical determination of the expression of cannabinoid receptors. Interesting data were obtained on changes in the CB1 receptors’ expression in rat brainstem after the introduction of cannabinoids and peptides of the Tyr-MIF-1 family against a background of heat stress. The parallel reporting of in vivo effects and specific in vitro changes in the expression of a given receptor would enable more precise conclusions regarding the role of the receptor, and the mediator system, respectively, for the observed effects. It would be interesting to track possible receptor changes (up-/downregulation, conformation shifts, etc.) under conditions of chronic (cold or other type of) stress. Furthermore, since receptors’ affinity is also important for the obtained effects, the determination of specific changes could enable a more thorough and detailed interpretation of the in vivo results.

Our research involves mainly acute trials. In the longer term, it would be interesting to follow up on the in vivo effect of exposing the animals to chronic stress. Various chronic stress setups have been described in the literature, and we also have some as yet unpublished data from the exposure of animals to repetitive swimming stress. Our preliminary results are concordant with the literature data that acute and chronic stress can differently affect some parameters [83,84]. On the other hand, studies on the effects of chronic stress are of particular interest, due to their clear connection with neurodegenerative diseases—dementia, Alzheimer’s disease, etc. [85,86,87]. Another interesting direction would be to establish the effect of another type of stress—in this sense, there is evidence that restraint stress in rats can be considered as the equivalent of psychosocial stress in humans [88], and other suitable models would be learned helplessness, social defeat, and social isolation [89]. Tracking the interactions between the cannabinoid and other mediator systems, e.g., dopaminergic and GABA-ergic, is also among our future, given the involvement of D1- and GABA_A_- receptors in the development of depressive behavior [90].

Our research was primarily aimed at the interpretation of the stress response, but the analysis of the literature data carried out in connection with a previous publication [53] suggested that the findings could be useful in the drug development field [91,92]. Both systems represent targets for pharmaceutical development: several agonist drugs are known [93,94] for both the cannabinoid and the serotonin receptors, since both the up- and downregulation of ECS-/5-HT-mediated signaling are desired in specific pathophysiological conditions [91,95]. Other drugs (e.g., opioids) antagonize the serotonin transporter and increase serotonin levels, causing so-called serotonin toxicity [96]. It is also important that CB receptors exhibit constitutive activity [97,98], meaning that their ligands’ intrinsic activities vary from agonist, through partial agonist and antagonist, to inverse agonist. Moreover, they exhibit biased signaling, thus structurally diverse agonists stabilize different ranges of active conformations of the receptors, consequently allowing the activation of different biochemical pathways [99]. Knowing the outcome of the exogenous antagonization/potentiation of a certain type of receptor, as well as the possible interactions between the different types, would provide certain guidelines for the therapeutic effect of the developed chemical structures. Also, the availability of data on specific interactions between individual receptors should be considered in view of possible adverse drug effects [95].

The potential clinical significance of this type of research is determined by the two systems—the cannabinoid and the serotoninergic themselves. Cannabinoids have been known about since ancient times, but their modern presence in medical practice is compromised by evidence of adverse effects [100]. At the same time, a large-scale campaign for their “rehabilitation” is underway, considering the beneficial effects of cannabinoids in the reduction of chronic pain treatment [101], chemotherapy-induced nausea and vomiting [102], and for some other medical conditions [103]. With this in mind, any study involving ECS contributes to elucidating its involvement in physiological and pathophysiological responses, thereby confirming or refuting the health benefits of cannabinoid-based substances. On the other hand, in recent years, the serotoninergic system has been the focus of numerous studies with the discovery of the kynurenine pathway [104]. In 2020, Savitz produced a rather interesting title [105], raising the question of the relationship of this system with major depressive disorder, bipolar disorder, and schizophrenia. The relationship between the serotonin and the kynurenine systems lies in the fact that they have a common precursor—tryptophan, and the development of mood disorders is associated with serotonin depletion. Therefore, any research on the interactions of the serotoninergic system with other systems can provide useful data and new perspectives to explore.

An additional benefit of our study is the thermal factor—in recent years, our team has investigated changes in the stress response at different environmental temperatures, and the results [37,38], as well as some not yet published, show that high and low temperatures differently affect stress reaction, which should be taken into consideration in the field of occupational medicine and health promotion programs for temperature-challenged workplaces.

We hope that the present study contributes to a better understanding of the role of the endogenous cannabinoid and the serotonergic systems interacting in both the pathogenesis and mediation of the stress response. Since both types of receptors (cannabinoid and serotonin) are widely distributed in the human body and, at the same time, represent valuable targets for the pharmacological influencing of a number of pathological conditions, we believe that the proposed information could provide interesting directions for different fields of science.

In conclusion, we have found a different type of interaction between the ECS and the serotoninergic system before and after stress. We assume that the potentiation of c-SIA (observed in the pre-stress treatment) is due to a higher degree of the effect of the serotonergic system that “maintains” analgesia, while the c-SIA (observed in the post-stress treatment) is more the result of the cannabinoids’ modulative effect on the HPA axis.

## Figures and Tables

**Table 1:** Summarizes the most important points of the results.

	Before 1 h Cold Stress	After 1 h Cold Stress
AEA+DPAT	transient potentiating of c-SIA on the 10th min; tendency to decrease c-SIA after the 10th min; stable level of c-SIA from the 30th min until the end of the time estimated.	tendency to decrease c-SIA from the 10th min; control values are reached soon after the 30th min; tendency to hyperalgesia on 40th min until the end of the experiment.
AEA+NAN	total abolishment of c-SIA	time-constant c-SIA
AM+DPAT	stable level of c-SIA	reduced c-SIA to the control values after the 20th min

AEA—exogenously administered anandamide; DPAT—5-HT1A-agonist; NAN—5-HT1A-antagonist; AM—CB1-antagonist.

## Data Availability

The protocols for the experiments carried out on the project are stored in the personal database of the leading researcher Hristina Nocheva, whom the Grant 85/04.06.2021, as well as the Permission Protocol from BFSA, were awarded.
